# Sports celebrity traits and millennial purchasing intentions: a cross-platform mixed-methods study

**DOI:** 10.3389/fspor.2025.1747465

**Published:** 2026-01-26

**Authors:** Dina El-Shihy

**Affiliations:** School of Business and Finance Marketing Department, NewGiza University (NGU), Giza, Egypt

**Keywords:** celebrity endorsement, endorser personality traits, millennial consumer behavior, mixed-methods research, perceived product quality, purchase intention, social media marketing, sports celebrities

## Abstract

**Introduction:**

This study examines how sports celebrities' personality traits—likability, expertise, credibility, and physical attractiveness—affect millennials' purchasing decisions across Instagram, Snapchat, and Twitter. Addressing a gap in prior endorsement research that often treats social media platforms as interchangeable, the study adopts a cross-platform perspective to investigate whether the effectiveness of celebrity traits varies by platform.

**Methods:**

A mixed-methods approach was employed. Quantitative data were collected through a survey of 228 millennials using stratified random sampling, while qualitative insights were obtained from semi-structured interviews with 12 participants. The interviews explored motivations, engagement patterns, and factors contributing to endorsement effectiveness. The study was guided by Source Credibility Theory, the Elaboration Likelihood Model, the Cognitive Response Model, and the Attitude-Toward-the-Ad Model.

**Results:**

The findings indicate that perceived product quality partially mediates the relationship between sports celebrities' personality traits and purchase intention. Moreover, the influence of each trait varies significantly across Instagram, Snapchat, and Twitter, suggesting that celebrity endorsement effectiveness is contingent on the social media platform.

**Discussion:**

By explicitly comparing multiple social media platforms and integrating quantitative and qualitative evidence, this study advances endorsement research by demonstrating that celebrity trait effectiveness is platform-specific rather than uniform. While all examined traits shape consumer responses, their relative importance differs across platforms, highlighting the need for platform-sensitive endorsement strategies and careful selection of celebrity endorsers. This cross-platform, mixed-method perspective provides nuanced insights into how digital celebrities influence consumer behavior and offers practical recommendations for marketers seeking to optimize influencer strategies across diverse social media channels.

## 1 Introduction

In the digital age, social media has revolutionized how people interact, share information, and promote brands, offering an unparalleled platform for engagement and communication (1, 2). Among the various strategies in social media marketing, celebrity endorsements stand out, significantly shaping consumer attitudes and purchase behaviors, particularly among millennials (3).

A notable trend is the strategic use of sports celebrities in endorsements, leveraging their athletic achievements, fame, and compelling personalities to influence consumers. These figures command significant attention, particularly from millennials, by actively sharing experiences and endorsing brands, making them effective allies for companies seeking to enhance brand visibility and drive consumer interest (4, 5).

Millennials, a prominent consumer demographic, are profoundly influenced by social media and its content (6–8). Research on how sports celebrities affect their preferences across platforms like Instagram, Snapchat, and Twitter is therefore crucial. Each platform appeals to millennials in distinct ways: Instagram's visual focus highlights physical appeal and lifestyle traits, Snapchat's ephemeral content fosters authenticity and trust, and Twitter's immediacy encourages direct engagement and textual credibility (9–11).

Despite growing research on celebrity endorsements, little is known about how specific personality traits—likability, expertise, credibility, and physical attractiveness—affect millennial purchasing intentions across these platforms. While attributes such as authenticity, charisma, and relatability contribute to endorsement effectiveness, they have not been systematically examined across platform contexts (12–14). Drawing on Source Credibility Theory, the Elaboration Likelihood Model (ELM), the Cognitive Response Model, and the Attitude-Toward-the-Ad Model, this study proposes that celebrity traits influence perceived product quality, which in turn shapes purchasing intentions. By focusing on Instagram, Snapchat, and Twitter—platforms with unique user demographics and content structures—this study explores these dynamics to offer actionable insights for marketers.

This research evaluates the impact of likability, expertise, credibility, and physical attractiveness, along with the mediating role of perceived product quality, on millennial purchasing decisions. In addition to quantitative survey data, qualitative interviews were conducted to explore participants' deeper perceptions, emotional responses, and reasoning. By integrating quantitative and qualitative insights, the study provides a richer understanding of how sports celebrity endorsements shape millennial consumer behavior and offers practical guidance for optimizing marketing strategies and strengthening brand engagement (12, 15).

## 2 Literature review

Celebrity endorsements involve collaboration between brands and public figures to promote products or services, relying on the celebrity's popularity and credibility to elevate brand appeal. Key traits such as likability, expertise, credibility, and physical attractiveness significantly influence endorsement success (16–18).

The influence of celebrity endorsements has evolved with social media, where platforms offer celebrities dynamic ways to engage their audiences. By sharing authentic content and real-life insights, they build deeper connections with followers, enhancing the impact of their endorsements (4, 19).

Sports celebrities, known for their athletic prowess and widespread recognition, uniquely shape the digital marketing landscape (20). Their ability to transcend the sports arena and connect with diverse audiences makes them valuable marketing assets. Platforms like Instagram, Snapchat, and Twitter cater to millennial preferences for authenticity, visual storytelling, and real-time interaction (21–23).

Moreover, while much of the existing literature focuses on quantitative evaluations of endorsement effectiveness, this study adopts a mixed-methods approach, combining survey-based analysis with qualitative interviews. The interviews enable in-depth exploration of the emotional and cognitive responses elicited by celebrity traits, providing contextual richness that complements the quantitative findings (24, 25).

### 2.1 Theoretical foundation

The theoretical underpinnings of this study rely on key frameworks that explain how celebrity personality traits influence perceived product quality and purchasing intentions, and why these effects vary across social media platforms.

Source Credibility Theory posits that an endorser's persuasiveness depends on expertise, trustworthiness, and likability (26–28). In this study, likability, expertise, credibility, and physical attractiveness represent facets of source credibility shaping consumer evaluations. A celebrity with high expertise enhances perceived product quality, increasing purchase intentions. Similarly, likable and credible celebrities foster positive affect, supporting product-related decisions.

Elaboration Likelihood Model (ELM) explains how consumers process persuasive messages via central or peripheral routes, depending on involvement and cognitive capacity (28–30). On social media, peripheral cues such as likability, attractiveness, and charisma often dominate, as millennials frequently consume content quickly and impressionistically. This lens supports the hypothesis that traits like likability and attractiveness influence both perceived product quality and purchase intentions, especially when consumers do not deeply scrutinize product details.

Cognitive Response Model suggests that consumers actively process endorsement messages, generating cognitive and affective responses that shape attitudes and behavior (31, 32). Perceived product quality is a key cognitive response: when a celebrity demonstrates expertise or authenticity, consumers interpret the product as reliable and valuable, influencing purchase intentions. This model provides the rationale for including perceived product quality as a mediator between celebrity traits and purchasing decisions.

Attitude-Toward-the-Ad Model emphasizes that favorable attitudes toward advertisements strengthen the link between endorsements and purchase intentions (33). In social media, engaging and authentic content amplifies the persuasive impact of celebrity traits. For instance, emotional engagement with a likable celebrity fosters positive attitudes toward the product, enhancing the effects of credibility and attractiveness on consumer behavior.

Platform-specific mechanisms further clarify these dynamics. On visually rich platforms like Instagram, peripheral cues such as likability and attractiveness dominate, enhancing perceived product quality and purchase intentions. Twitter's text-oriented environment emphasizes central route processing, where expertise and credibility drive cognitive evaluations. Snapchat's informal and ephemeral content facilitates quick trust-building and engagement, leveraging a combination of likability and credibility.

Taken together, these frameworks provide a coherent rationale for hypothesizing how celebrity personality traits influence perceived product quality and purchasing intentions across Instagram, Snapchat, and Twitter. Source Credibility Theory explains why traits such as likability, expertise, credibility, and attractiveness enhance message acceptance, elevating perceived product quality—a key precursor to behavioral intention. ELM shows that millennials often rely on peripheral cues, making personality traits especially impactful in shaping perceived quality. The Cognitive Response Model clarifies that consumers generate cognitive evaluations based on affective and cognitive impressions of the celebrity. Finally, the Attitude-Toward-the-Ad Model supports the role of perceived product quality as a mediator, as consumers' evaluations of the message and the celebrity shape beliefs about the product, influencing purchase decisions.

These theories also guide interpretation of qualitative data, providing a basis for understanding how consumers describe experiences with celebrity endorsements, including perceptions of trust, expertise, and appeal. By examining how traits such as likability and credibility affect millennial purchasing intentions, this study uncovers nuanced mechanisms driving successful endorsements on Instagram, Snapchat, and Twitter. The mediating role of perceived product quality underscores the importance of aligning endorsements with consumer expectations to optimize marketing outcomes.

### 2.2 Hypotheses development

Likability plays a pivotal role in the success of celebrity endorsements, enabling endorsers to forge positive associations with the products they promote. Consumers often perceive products endorsed by likable celebrities as higher quality due to the endorser's personal appeal (17). This is especially relevant on visually driven platforms like Instagram, where relatability and personal engagement are paramount. Millennials are drawn to sports celebrities who project warmth and authenticity on social media, creating a sense of trust and personal connection that can translate to the endorsed product (20, 34). For instance, a sports celebrity sharing glimpses of their personal life and interacting with fans is more likely to enhance the perceived quality and desirability of the products they endorse (35). Likable celebrities not only foster trust but also elevate the attractiveness and credibility of the associated products, shaping millennial purchasing behavior (36–38). According to ELM, Instagram's visually rich and peripheral cue–dominated environment amplifies the impact of likability, whereas Snapchat leverages informal and spontaneous interactions to reinforce trust. Twitter relies less on peripheral cues, emphasizing quick impressions rather than detailed engagement. Accordingly, based on Source Credibility Theory and ELM, likability serves as a persuasive cue that increases message acceptance and influences the cognitive evaluation of the product, thereby strengthening both perceived quality and purchasing intentions.

- *H1: Sports celebrity likability positively influences millennial purchasing intentions on social media platforms (a. Instagram, b. Snapchat, c. Twitter).*
- *H2: Sports celebrity likability positively influences perceived product quality on social media platforms (a. Instagram, b. Snapchat, c. Twitter).*

Expertise is another critical dimension of celebrity endorsements. Endorsers perceived as knowledgeable and authoritative are often regarded as credible, thereby positively shaping consumer attitudes and purchasing intentions (39, 40). This credibility is strengthened when celebrities offer detailed, firsthand insights about the products they endorse, reinforcing consumer trust (41). For example, a professional athlete endorsing sports equipment or sharing technical knowledge about a product's features can resonate deeply with millennials, who value informed opinions and substantive content (42–44). Based on the Cognitive Response Model, Twitter's text-focused environment facilitates central route processing, making expertise especially impactful, while Instagram and Snapchat benefit from visual demonstrations of expertise. Consequently, the influence of expertise enhances quality-related beliefs that directly affect purchase intention.

- *H3: Sports celebrity expertise positively influences millennial purchasing intentions on social media platforms (a. Instagram, b. Snapchat, c. Twitter).*
- *H4: Sports celebrity expertise positively influences perceived product quality on social media platforms (a. Instagram, b. Snapchat, c. Twitter).*

Credibility encompasses the trustworthiness and reliability attributed to a celebrity endorser. It shapes consumer attitudes by enhancing confidence in the endorsed products (45, 46). Authenticity, demonstrated through genuine testimonials and transparent communication, is particularly influential on platforms like Twitter, where users value openness and honesty (47). Millennials are likely to trust endorsements from credible sports celebrities, especially when the endorser's reputation aligns with their values. Source Credibility Theory suggests that Snapchat's informal interactions allow credibility to be communicated quickly through repeated exposure and casual engagement, whereas Instagram conveys credibility through consistent, authentic storytelling, and Twitter emphasizes textual evidence of trustworthiness. Source Credibility Theory positions trustworthiness as a central driver of message acceptance, making credibility a direct antecedent of both perceived product quality and purchasing intention.

- *H5: Sports celebrity credibility positively influences millennial purchasing intentions on social media platforms (a. Instagram, b. Snapchat, c. Twitter).*
- *H6: Sports celebrity credibility positively influences perceived product quality on social media platforms (a. Instagram, b. Snapchat, c. Twitter).*

Physical attractiveness, characterized by a celebrity's aesthetic appeal, plays a vital role in capturing consumer attention and enhancing the memorability of endorsements (48). Platforms like Instagram, with their emphasis on visuals, heighten the impact of attractive endorsers. For instance, millennials may be more inclined to associate a physically fit and appealing sports celebrity with high-quality and prestigious products (49, 50). These associations influence product desirability and consumer attitudes. Consistent with ELM, physical attractiveness functions as a peripheral cue, with Instagram highlighting visual appeal, Snapchat leveraging attractiveness in informal story content, and Twitter relying more on cognitive evaluation than aesthetic cues.

- *H7: Sports celebrity physical attractiveness positively influences millennial purchasing intentions on social media platforms (a. Instagram, b. Snapchat, c. Twitter).*
- *H8: Sports celebrity physical attractiveness positively influences perceived product quality on social media platforms (a. Instagram, b. Snapchat, c. Twitter).*

Perceived product quality acts as a crucial mediator between celebrity traits and consumer behavior. It reflects consumers' subjective evaluations of a product's reliability, performance, and overall appeal (51). Endorsements by likable, credible, and attractive sports celebrities can enhance perceptions of product quality, fostering greater purchase intent. For instance, millennials may associate a celebrity's expertise or aesthetic appeal with attributes such as durability and prestige, increasing their willingness to buy (52, 53). This mediating role aligns directly with the Attitude-Toward-the-Ad Model and the Cognitive Response Model, and incorporates platform-specific mechanisms where Instagram emphasizes peripheral cues, Twitter emphasizes central processing, and Snapchat balances informal engagement with trust cues.

- *H9: Perceived product quality positively influences millennial purchasing intentions on social media platforms (a. Instagram, b. Snapchat, c. Twitter).*
- *H10: Perceived product quality mediates the relationship between celebrity likability and purchasing intentions on social media platforms (a. Instagram, b. Snapchat, c. Twitter).*
- *H11: Perceived product quality mediates the relationship between celebrity credibility and purchasing intentions on social media platforms (a. Instagram, b. Snapchat, c. Twitter).*
- *H12: Perceived product quality mediates the relationship between celebrity expertise and purchasing intentions on social media platforms (a. Instagram, b. Snapchat, c. Twitter).*
- *H13: Perceived product quality mediates the relationship between celebrity physical attractiveness and purchasing intentions on social media platforms (a. Instagram, b. Snapchat, c. Twitter).*

**Figure 1:**
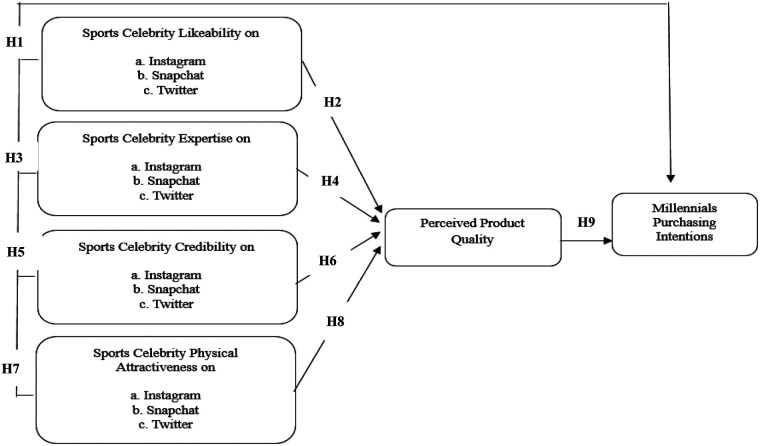
*Conceptual framework*. Source: Developed by the authors.

Table 1 summarizes existing studies on sports celebrity endorsements and highlights the research gaps addressed by the present study. While prior research examined general effects of celebrity traits or consumer behavior, few studies investigated trait-specific influences across multiple platforms or incorporated qualitative insights. This study fills these gaps by combining cross-platform SEM analysis with interviews, linking personality traits—likability, expertise, credibility, and physical attractiveness—to perceived product quality and millennial purchasing intentions (Figure 1).

**Table 1:** Comparison of existing research on celebrity endorsements and research gaps addressed by the present study.

Study	Major findings	Research gap addressed by this study
Ferreira et al. (54)	Sports celebrities' marketable lifestyles enhance consumer engagement; brand-celebrity congruence plays a critical role.	Did not examine how specific personality traits (likability, credibility, expertise, attractiveness) operate across different social media platforms. This study explicitly investigates trait effects on Instagram, Snapchat, and Twitter.
Baltezarević & Papakonstantinidis (55)	Reputation of sports celebrities impacts brand standing; inappropriate behavior poses risks.	Prior work overlooks platform-specific variations in trait perception. This study explores how traits differentially influence perceived product quality depending on platform features.
El-Shihy (56)	Psychological mechanisms, including personality traits, affect consumer engagement with celebrity endorsements.	Focuses only on quantitative or general mechanisms; does not combine qualitative insights to explain why consumers respond differently. This study uses a mixed-methods approach to capture both statistical effects and consumer interpretations.
Kang et al. (57)	Celebrity traits influence brand defending and purchase intent among consumers.	Examines behavioral outcomes but lacks cognitive and emotional depth. This study adds qualitative insight to understand how and why traits drive purchase intention across platforms.
Shezi (58)	Brand personality of sports celebrities boosts identification and purchase intention.	Limited to overall brand personality; does not dissect individual traits or consider platform context. This study provides trait-specific, platform-focused analysis to clarify underlying mechanisms.
Shao et al. (59)	Celebrity endorsement influences impulsive buying; credibility and trait alignment are critical.	Focuses on impulsive buying, not perception of product quality or cross-platform differences. This research evaluates both product perception and intention across multiple platforms.
Hussain et al. (60)	Personality congruence between consumers and celebrities affects endorsement outcomes across platforms.	Explores congruence broadly but lacks mixed-methods depth and detailed examination of each platform's characteristics. This study combines SEM and interviews to explain platform-specific variations.
Bush et al. (61)	Perceived familiarity and identification influence Generation Y's endorsement responses.	Historical perspective; does not address modern social media platforms or platform-specific mechanisms. This study updates context for millennials using Instagram, Snapchat, and Twitter, considering both quantitative and qualitative insights.

Source: Developed by the authors.

## 3 Methods

### 3.1 Research design and sampling

This study adopted a mixed-methods sequential explanatory design (88), in which quantitative data collection and analysis were followed by qualitative interviews to provide deeper insight into survey findings. Quantitative results guided the development and focus of the interviews, allowing for rich integration of participants' perceptions of sports celebrity endorsements with the Structural Equation Modeling (SEM) results.

A cross-sectional survey was conducted among Egyptian millennials aged 23–41 who actively use Instagram, Snapchat, and Twitter. Because participants were recruited via online platforms (Google Forms) and social media networks, a true probability sampling frame was not feasible. A stratified quota sampling approach was adopted to approximate representativeness across platforms. Strata were defined by participants' primary social media platform usage. Recruitment involved sharing the survey link through social media posts, university networks, and snowballing. Participants were not randomly selected from a master list; rather, quota targets ensured adequate numbers for each platform. Out of the final 228 respondents, 80 were from Instagram, 75 from Snapchat, and 73 from Twitter. Participants could use multiple platforms, with 60% reporting multi-platform usage.

Inclusion criteria required participants to be aged 23–41, reside in Egypt, and actively use at least one of the target platforms. Exclusion criteria were non-users or participants outside the age range. The minimum sample size was determined using the formula suggested by Nyalungwe et al. (62):

$$n=\left(z^{2}\times p\times (1-p)\right)/e^{2}=\left(\left(1.96{)}^{2}\times 0.5\times (1-0.5\right)\right)/(0.1{)}^{2}\approx 96.04\Rightarrow n=97<228$$

The achieved sample of 228 exceeded the minimum required for analysis. Self-selection bias may have influenced responses. A conventional response rate cannot be calculated due to the open distribution method, which is discussed as a limitation in Section 7.

### 3.2 Instrumentation

A structured questionnaire was developed using validated scales from prior literature, with items adapted to the context of sports celebrity endorsements on social media. The questionnaire comprised four sections. The first collected demographic information, including age, gender, and social media usage patterns. The second assessed sports celebrity personality traits—likability, expertise, credibility, and physical attractiveness—using items adapted from McCormick (63). Items were adjusted to refer specifically to social media posts.

The third section measured perceived product quality, based on Akoglu and Özbek (64), with items reflecting consumer perceptions after exposure to social media content shared by sports celebrities. The fourth section focused on purchasing intentions, adapted from Priyankara et al. (65), capturing the likelihood of purchase following social media exposure. All measurement items, response anchors (1 = strongly disagree to 5 = strongly agree), and their sources are provided in Appendix A, with modifications clearly documented.

### 3.3 Quantitative data collection and analysis

The quantitative survey was distributed via Google Forms over a one-month period using social media posts, university networks, and snowballing. Procedural remedies reduced common method bias, including anonymous responses, mixing item order, and separating predictors from outcomes.

Data were analyzed using SPSS for descriptive statistics and Partial Least Squares Structural Equation Modeling (PLS-SEM) to examine relationships among latent constructs. PLS-SEM was chosen over CB-SEM due to the moderate sample size (*N* = 228) and model complexity, which included multiple latent constructs, mediating paths, and multigroup comparisons. Reliability and validity were assessed using Cronbach's Alpha, Composite Reliability (CR), Average Variance Extracted (AVE), and factor loadings. Discriminant validity was evaluated with both Fornell-Larcker and HTMT methods. Multigroup analysis was conducted based on participants' primary platform (Instagram, Snapchat, Twitter), with the Welch–Satterthwaite test complementing PLS-MGA to identify significant differences in path coefficients across groups.

### 3.4 Qualitative phase

Semi-structured interviews were conducted with 12 participants purposively selected from the survey sample to ensure variation in gender, engagement level, and primary platform (four participants per platform). Participants' ages ranged from 25 to 32 years, reflecting the millennial target group. Data saturation was reached when no new themes emerged.

Interviews explored participants' perceptions of celebrity traits, platform-specific content, emotional reactions, and the influence of endorsements on perceived product quality and purchase intention. Questions covered both general impressions and platform-specific perceptions, including engagement with content, emotional responses, and judgments of authenticity, expertise, credibility, and attractiveness. Main topics and example prompts guided the discussion to ensure coverage of areas relevant to the hypotheses (see Appendix A2 for the full interview guide).

Transcripts were analyzed using thematic analysis (89). Open coding identified meaningful data segments, axial coding grouped codes into categories, and selective coding identified overarching themes. Coding reliability was ensured through careful review and discussion by the research team, with examples of how raw statements were transformed into codes, categories, and themes provided in Appendix A3 for methodological transparency.

Qualitative findings were explicitly integrated with SEM results to confirm and nuance specific relationships, providing richer context for platform-specific variations. Integration strengthened the mixed-methods interpretation.

### 3.5 Ethical considerations

Participant data were handled anonymously to ensure confidentiality. All participants received explanations of the study objectives, procedures, and voluntary participation requirements. Informed consent was obtained, and ethical approval was granted by the Institutional Review Board (IRB) at Badr University in Cairo (BUC).

## 4 Results

### 4.1 Quantitative findings

#### 4.1.1 Descriptive analysis

The demographic profile of the 228 respondents is summarized in Table 2. The sample consisted of 136 males (59.65%) and 92 females (40.35%), offering a reasonably balanced gender distribution. The largest age group was 23–27 years (31.14%), followed by 27–32 years (29.39%). Most participants (91.67%) held a bachelor's degree, indicating a highly educated sample. Income distribution showed that 37.72% earned between 20,000 and 30,000 EGP, suggesting a predominantly middle-income demographic.

**Table 2:** Frequency table for demographics.

Variable	Category	Frequency	Percentage
Gender	Female	92	40.35
	Male	136	59.65
Age	23–27	71	31.14
	27–32	67	29.39
	32–36	43	18.86
	36–41	47	20.61
Education	High School	3	1.32
	Bachelor's degree	209	91.67
	Postgraduate Degree	16	7.01
Income	<10,000 EGP	40	17.54
	10,000–20,000 EGP	66	28.95
	20,000–30,000 EGP	86	37.72
	>30,000 EGP	36	15.79

Source: Calculations based on sample collected through surveys using SPSS software.

#### 4.1.2 Confirmatory factor analysis (CFA)

To assess measurement validity, Confirmatory Factor Analysis (CFA) was conducted. Common Method Bias (CMB) was addressed using multiple approaches: all VIF values were below 5, indicating no substantial multicollinearity (66). Procedural remedies were also applied, including anonymous survey responses, mixing the order of items, and separating predictor and outcome constructs to reduce method bias.

Reliability and convergent validity were assessed using Cronbach's Alpha, Composite Reliability (CR), Average Variance Extracted (AVE), and item-level outer loadings. As shown in Table 3, all constructs—including Likability (LIK), Expertise (EXP), Credibility (CRD), Physical Attractiveness (PAT), Perceived Product Quality (PPQ), and Purchase Intention (PI)—met the recommended thresholds for reliability and convergent validity (*α* > 0.70; CR > 0.70; AVE > 0.50). Each item's outer loading exceeded the 0.70 benchmark, confirming that all items contributed meaningfully to their respective constructs. The “Status” column indicates that all items were retained for further analysis, ensuring consistency with the survey instrument and alignment with SEM and multigroup results.

**Table 3:** Reliability and validity metrics (CFA).

Construct	Item	Outer Loading	Cronbach's Alpha	CR	AVE	Status
Likability (LIK)	LIK1	0.812	0.82	0.88	0.61	Retained
	LIK2	0.788				Retained
	LIK3	0.801				Retained
Expertise (EXP)	EXP1	0.845	0.84	0.90	0.63	Retained
	EXP2	0.811				Retained
	EXP3	0.829				Retained
Credibility (CRD)	CRD1	0.802	0.80	0.87	0.60	Retained
	CRD2	0.788				Retained
	CRD3	0.793				Retained
Physical Attractiveness (PAT)	PAT1	0.782	0.78	0.85	0.58	Retained
	PAT2	0.769				Retained
	PAT3	0.771				Retained
Perceived Product Quality (PPQ)	PPQ1	0.831	0.83	0.89	0.62	Retained
	PPQ2	0.819				Retained
	PPQ3	0.825				Retained
Purchase Intention (PI)	PI1	0.814	0.81	0.88	0.61	Retained
	PI2	0.805				Retained
	PI3	0.808				Retained

Discriminant validity was assessed using the Fornell-Larcker criterion and Heterotrait-Monotrait (HTMT) ratios. All constructs satisfied Fornell-Larcker criteria (square roots of AVE exceeded inter-construct correlations) and HTMT values were below 0.90, confirming satisfactory discriminant validity.

PLS-SEM was employed instead of CB-SEM due to the moderate sample size (*N* = 228) and the model complexity, which included multiple latent constructs, mediating paths, and multigroup comparisons. PLS-SEM is robust under these conditions and supports both predictive and exploratory analysis (90).

#### 4.1.3 Structural equation model (SEM)

Table 4 summarizes the direct and indirect effects of celebrity traits on perceived product quality and purchase intention. The SEM results confirm significant effects of all celebrity traits on perceived product quality and purchase intention, with partial mediation through product quality.

**Table 4:** Summary of direct and indirect effects of celebrity traits on product quality and purchase intention.

Path	Direct to Perceived Product Quality	Direct to Purchase Intention	Indirect via Product Quality
Likeability	0.398**	0.385**	0.363***
Expertise	0.466***	0.446***	0.491***
Credibility	0.483***	0.301***	0.375***
Physical attractiveness	0.342***	0.386***	0.399***
Perceived product quality → purchase intention	—	0.409***	—

Source: Based on Calculations using Smart PLS.

Note: ***, **, and * denote statistical significance at the 1%, 5%, and 10% levels, respectively.

#### 4.1.4 Multigroup analysis

Although multigroup analysis and Welch–Satterthwaite tests were conducted to assess differences in path coefficients across platforms, no formal measurement invariance testing (e.g., MICOM) was performed. Cross-group comparisons should therefore be interpreted cautiously, and future studies could include MICOM or other invariance assessments. Groups were defined based on participants' primary social media platform (Instagram, Snapchat, Twitter). Sample sizes were 80 for Instagram, 75 for Snapchat, and 73 for Twitter. Participants could report multi-platform use, but the primary platform determined their group assignment. Table 5 presents the direct effects by platform, while Table 6 presents the mediating effects. The multigroup analysis revealed distinct platform-specific patterns in how celebrity traits influenced consumer responses.

**Table 5:** Multi-group analysis of direct effects by platform.

Path	Instagram (Original Sample)	Instagram (Std. Error)	Snapchat (Original Sample)	Snapchat (Std. Error)	Twitter (Original Sample)	Twitter (Std. Error)
Likeability → perceived product quality	0.565**	0.243	0.241***	0.05	0.323*	0.181
Likeability → purchase intention	0.286**	0.076	0.609**	0.245	0.114*	0.052
Expertise → perceived product quality	0.577*	0.305	0.401***	0.15	0.704***	0.213
Expertise → purchase intention	0.138**	0.055	0.386**	0.151	0.577***	0.194
Credibility → perceived product quality	0.607**	0.278	0.347**	0.137	0.305**	0.103
Credibility → purchase intention	0.241*	0.136	0.36*	0.176	0.128***	0.023
Physical attractiveness → perceived product quality	0.141	0.231	0.046	0.163	0.511*	0.305
Physical attractiveness → purchase intention	0.263	0.267	0.192	0.136	0.253*	0.112
Perceived product quality → purchase intention	0.263*	0.154	0.312***	0.105	0.41***	0.099

Source: Based on calculations using smart PLS.

Note: ***, **, and * denote statistical significance at the 1%, 5%, and 10% levels, respectively.

**Table 6:** Multi-group analysis of mediating effects by platform.

Path	Instagram (Original Sample)	Instagram (Std. Error)	Snapchat (Original Sample)	Snapchat (Std. Error)	Twitter (Original Sample)	Twitter (Std. Error)
Likeability → perceived product quality → purchase intention	0.149***	0.015	0.075***	0.022	0.132**	0.049
Expertise → perceived product quality → purchase intention	0.152***	0.040	0.125*	0.067	0.289**	0.115
Credibility → perceived product quality → purchase intention	0.170*	0.101	0.108*	0.060	0.140***	0.029
Physical attractiveness → perceived product quality → purchase intention	0.037	0.070	0.0144	0.057	0.210*	0.115

Source: Based on Calculations using Smart PLS.

Note: ***, **, and * denote statistical significance at the 1%, 5%, and 10% levels, respectively.

The following discussion highlights primary patterns across platforms, with detailed numerical results available in Tables 5–7, including path coefficients and Welch–Satterthwaite test statistics. The multigroup analysis revealed distinct platform-specific patterns in how celebrity traits influenced consumer responses. Likability had the strongest effect on purchase intention on Snapchat (*β* = 0.609), reflecting the platform's informal and peer-driven environment, where relatable traits tend to resonate strongly with users. Expertise emerged as the dominant trait on Twitter, showing the greatest impact on perceived product quality (*β* = 0.704) and purchase intention (*β* = 0.577), aligning with the platform's information-oriented nature, where credibility and knowledge are especially valued. Physical attractiveness was only significant on Twitter, suggesting that users engage more selectively with visually appealing content in this context. The effects of credibility varied across platforms, emphasizing differences in how users respond to visual appeal versus peer validation. These findings underscore the importance of developing endorsement strategies that are tailored to the characteristics and expectations of each social media platform.

**Table 7:** Welch-Satterthwaite test comparing path coefficients across platforms.

Path	Instagram vs Snapchat	Instagram vs Twitter	Snapchat vs Twitter
Likeability → perceived product quality	0.324***	0.242***	−0.082*
Likeability → purchase intention	−0.323***	0.172***	0.495**
Expertise → perceived product quality	0.176***	−0.127***	−0.303**
Expertise → purchase intention	−0.248***	−0.439**	−0.191***
Credibility → perceived product quality	0.26***	0.302**	0.042
Credibility → purchase intention	−0.119***	0.113***	0.232*
Physical attractiveness → perceived product quality	0.095**	−0.37***	−0.465**
Physical attractiveness → purchase intention	0.071*	0.01	−0.061
Perceived product quality → purchase intention	−0.049	−0.147**	−0.098*

Source: Based on Calculations using Smart PLS.

Note: ***, **, and * denote statistical significance at the 1%, 5%, and 10% levels, respectively.

#### 4.1.5 Welch-Satterthwaite test

The Welch–Satterthwaite test was employed as a complementary approach to PLS-MGA and permutation tests to compare path coefficients across groups with unequal variances and sample sizes. This method helped identify statistically significant differences in the strength of relationships that may not be fully captured by PLS-MGA alone. The results in Table 7 indicate that expertise exerted a relatively more negative effect on purchase intention on Twitter compared to Instagram or Snapchat, suggesting that users on this platform are more critical or selective regarding expert endorsements. Additionally, physical attractiveness showed stronger negative effects when comparing Snapchat and Twitter, highlighting that the influence of visual appeal differs across platforms. These findings have important practical implications, indicating that endorsement strategies should emphasize the dominant celebrity trait for each platform and consider platform-specific user expectations.

#### 4.1.6 Model fit and predictive power

The R^2^ values indicate strong explanatory power (61.7% for perceived product quality; 57.9% for purchase intentions). Q^2^ values were positive across all constructs, confirming predictive relevance. The SRMR was 0.029, indicating excellent model fit (67).

### 4.2 Qualitative findings

To complement the quantitative analysis, qualitative data were gathered through semi-structured interviews with 12 millennial participants selected from the original survey pool. These interviews were designed to deepen understanding of how sports celebrity traits influence perceptions of product quality and purchase intention across Instagram, Snapchat, and Twitter. All interviews were transcribed verbatim and analyzed thematically, resulting in four key themes that support and extend the statistical findings.

#### 4.2.1 Authentic engagement enhances likability

Participants often linked likability to more than just physical appearance. They emphasized how a celebrity's genuine communication style and personal content shaped impressions, particularly on Instagram. Visual storytelling and casual, everyday moments helped build emotional connection.

- One male participant (primary platform: Instagram, age 27) explained: “*I follow a few athletes who post behind-the-scenes stuff—family dinners, workouts, even silly moments. That's when I start to feel like I know them. It's not just a brand talking—it's them.”*
- One female participant (primary platform: Instagram, age 26) shared: “*If I like the person, I’m more open to what they’re promoting—even if I hadn’t considered that product before.”*
- One male participant (primary platform: Snapchat, age 30) added: “*Even short stories or candid clips make me feel like I understand their personality. That makes me trust their recommendations more.”*
- One female participant (primary platform: Twitter, age 28) noted: “*Sometimes I don’t even care about the product at first. If I like the athlete, I’ll pay attention and might end up buying it.”*

These insights support the quantitative result showing that likability had the strongest influence on perceived product quality on Instagram (*β* = 0.565), highlighting that emotionally engaging content contributes to stronger brand association.

#### 4.2.2 Expertise must be contextual and demonstrated

Participants described expertise as effective when the celebrity demonstrated specific product knowledge—especially for performance-related items. Twitter was noted as a suitable platform for this, as it allows for detailed written content and product explanations.

- One male participant (primary platform: Twitter, age 31) noted: “*When a footballer breaks down how their boots help them pivot better or gives technical feedback on a supplement—it sticks. That's what I want to hear.”*
- One female participant (primary platform: Twitter, age 29) added: “*Twitter is where I go for insights. I don’t care if they look good—I care if they know what they’re talking about.”*
- One male participant (primary platform: Instagram, age 26) explained: “*Even on Instagram, I notice when they share tips or explain why they choose a product. That shows real expertise.”*
- One female participant (primary platform: Snapchat, age 27) said: “*I trust celebrities who show me exactly how they use a product. Their knowledge matters more than appearance.”*

These comments align with SEM findings showing expertise had the highest effect on purchase intention on Twitter (*β* = 0.577), emphasizing that platform features shape how traits like expertise are perceived.

#### 4.2.3 Trust and credibility are built through consistency

Credibility was tied to how consistently a celebrity aligned with the products they endorsed and whether they showed authentic behavior over time. Participants emphasized the value of transparency, especially on Snapchat, which they saw as more candid and less curated.

- One male participant (primary platform: Snapchat, age 30) explained: “*If I see someone pushing different brands every week, I stop trusting them. But when it's someone who's been with the same brand for a while—and shows how they use it—I take it seriously.”*
- One female participant (primary platform: Snapchat, age 25) added: “*Snapchat feels more real. You catch them off guard sometimes. That's where I judge if they actually believe in the product.”*
- One male participant (primary platform: Twitter, age 32) noted: “*I look at how consistent their endorsements are. If it matches their values and performance, I trust them more.”*
- One female participant (primary platform: Instagram, age 26) said: “*Credibility is about transparency. If they genuinely use the product, I feel confident following their advice.”*

These responses align with quantitative findings, where credibility significantly influenced purchase intention on Snapchat (*β* = 0.360), indicating that informal, real-time content can foster trust.

#### 4.2.4 Physical attractiveness is context-dependent and aspirational

While attractiveness was acknowledged as influential, participants emphasized that it was most effective when paired with performance, discipline, or relevance to the product. Twitter emerged as the platform where this blend of traits was most noticed.

- One male participant (primary platform: Twitter, age 31) shared*: “Yeah, looks catch attention—but if that person clearly trains hard and maintains discipline, I respect that. It tells me their product might reflect that same quality.”*
- One female participant (primary platform: Twitter, age 28) added: “*Attractiveness matters, but only when it connects with their performance. I’d buy protein powder from someone who looks like they use it—not just someone with a filter.”*
- One male participant (primary platform: Instagram, age 26) noted: “*On Instagram, attractiveness draws me in, but performance and authenticity keep me engaged.”*
- One female participant (primary platform: Snapchat, age 27) said: “*I notice effort and discipline more than looks, but the combination makes the endorsement feel credible.”*

These comments reinforce the quantitative result that physical attractiveness had the strongest effect on perceived product quality on Twitter (*β* = 0.511), highlighting the importance of relevance and context in interpreting this trait.

Overall, the qualitative themes offer emotional and interpretive depth to the statistical relationships found in the SEM model. They illustrate how personality traits—especially likability, expertise, credibility, and physical attractiveness—are understood and evaluated by consumers in platform-specific ways. These insights support the partial mediation role of perceived product quality, showing that consumer responses are shaped by both emotional connection and trait-based cues, which together inform millennial purchasing decisions across platforms.

## 5 Discussion

The positive association between sports celebrities' likability and millennial purchasing intentions across Instagram, Snapchat, and Twitter is consistent with earlier studies (68) and can be interpreted through ELM (91). According to this model, when consumers like a celebrity endorser, they are more likely to rely on surface-level cues, such as appearance or personality. For millennials, the presence of likable sports celebrities appears to foster positive emotions and associations, which are linked to greater interest in the endorsed product. This supports the view that likability functions as a heuristic cue that enhances the celebrity's appeal and is associated with consumers' product-related evaluations (49). Qualitative findings converge with these results, as participants frequently connected likability with emotional connection and relatability—especially on Instagram. Respondents reported feeling “*close”* to athletes who posted personal, lifestyle-focused content, which was associated with enhanced willingness to trust endorsements. Platform-specific differences were evident: Instagram's visual and lifestyle-oriented features facilitate emotional storytelling, strengthening likability, whereas Snapchat or Twitter content is often perceived as more transient or informational.

In a similar vein, the positive relationship between credibility and millennial purchasing intentions aligns with the Source Credibility Model (69) and recent research (70). Celebrities perceived as credible are commonly viewed as trustworthy and reliable, which may enhance the persuasiveness of their endorsements. This finding is consistent with prior studies highlighting credibility as a key component of endorsement effectiveness (71, 72). Interview participants reinforced these patterns, noting that credibility was closely tied to consistent behavior and long-term product use. Several respondents mentioned discontinuing trust in endorsers who “*promoted something different every week,”* underscoring the importance of perceived authenticity. Platform differences also emerged, as Snapchat's informal and real-time content enabled users to assess authenticity and identify inconsistencies, potentially amplifying the role of credibility in shaping purchase-related judgments.

Beyond credibility, the observed association between expertise and purchasing intentions is also consistent with the Source Credibility Model, which suggests that consumers place greater weight on recommendations from endorsers perceived as knowledgeable (69, 73). Expertise was linked to more favorable product evaluations and greater confidence in purchase decisions (74–76). Qualitative findings mirrored the quantitative results, particularly on Twitter, where participants valued detailed explanations and technical insights. These platform-specific patterns can be attributed to Twitter's text-oriented environment, which facilitates central-route processing and the communication of expertise more effectively than visually dominant or ephemeral platforms.

In contrast to cognitive traits, the influence of physical attractiveness was generally consistent with previous research, including findings associated with the halo effect (77, 78). Attractive celebrities tended to capture attention and generate favorable initial impressions, although this trait was not uniformly influential across platforms. Several interviewees associated attractiveness with effort, discipline, and authenticity—particularly on Twitter—suggesting that physical appeal may interact with cognitive evaluations rather than operate solely as a visual cue. These qualitative insights add nuance to the SEM results, showing that attractiveness effects are context-dependent rather than universal.

Taken together, these trait-based evaluations converged most strongly through perceptions of product quality. Perceived product quality emerged as a central construct associated with millennial purchasing intentions, supporting prior research emphasizing quality perceptions in consumer decision-making (79–81). Qualitative responses further clarified this relationship, as participants frequently linked perceived quality to observable product use, demonstrated reliability, or detailed explanations by the celebrity. Notably, some respondents reported refraining from purchase when endorsements appeared inconsistent, fake, or inauthentic, reinforcing the role of perceived product quality as an intervening mechanism between celebrity traits and purchase intentions.

The mediating role of perceived product quality aligns with the Cognitive Response Model (91), which emphasizes consumers' internal evaluations of endorsement messages. Participants described assessing whether a celebrity's lifestyle and personality aligned with the promoted product, with one noting: “*If they live the lifestyle they’re promoting, I believe the product works. If not, I scroll past”*. These qualitative insights complement the SEM findings, illustrating how cognitive evaluations are shaped by both emotional engagement and trait-based cues. Overall, the integration of quantitative and qualitative results highlights how celebrity traits, perceived product quality, and platform-specific affordances jointly shape millennial purchasing decisions, offering theoretically grounded and practically relevant implications for cross-platform endorsement strategies.

## 6 Implications

This research offers clear and original contributions by providing robust, mixed-method evidence on how sports celebrity endorsements shape millennial consumer behavior across distinct social media platforms. By integrating cross-platform SEM with qualitative interviews, the study moves beyond prior single-platform or trait-aggregated approaches and offers a more granular understanding of endorsement effectiveness. The findings generate actionable insights for scholars and practitioners by demonstrating not only which celebrity traits matter, but also the mechanisms through which they influence perceived product quality and purchasing intentions under platform-specific conditions.

The inclusion of qualitative perspectives further strengthens these contributions by explaining how and why consumers interpret endorsement cues differently across Instagram, Snapchat, and Twitter, thereby deepening the interpretive and explanatory understanding of the quantitative results.

### 6.1 Theoretical implications

This study advances theory-building by demonstrating that the influence of sports celebrity traits is platform-dependent rather than universal, thereby challenging earlier assumptions that endorsement strategies function similarly across social media environments. A key theoretical contribution lies in showing that traits such as likability (26, 27), expertise (82, 83), credibility (28, 84), and physical attractiveness (85, 86) exhibit varying levels of effectiveness depending on the platform through which endorsements are communicated. The qualitative findings reinforce this interpretation by revealing that Instagram's visual modality amplifies likability, Snapchat's informal and ephemeral environment heightens the importance of authenticity and relatability, and Twitter's text-driven format foregrounds expertise and informational value.

These findings highlight the need for future theoretical models of celebrity endorsement to explicitly incorporate platform context, including content modality, interaction norms, and platform-specific affordances. Rather than treating social media as a homogeneous communication environment, the results suggest that consumer evaluations are shaped by the functional purpose and interaction style of each platform, with users relying on different evaluative cues accordingly. This insight extends prior endorsement research by conceptualizing consumer engagement as a multi-platform and context-sensitive process.

In addition, this research extends existing frameworks such as the Cognitive Response Model (31, 87) by identifying perceived product quality as a central mechanism linking celebrity traits to purchasing intentions. While earlier studies acknowledged cognitive evaluations in endorsement effectiveness, they often paid limited attention to how product quality perceptions emerge from trait-based cues. The present findings, supported by both SEM results and qualitative insights, indicate that perceived quality integrates emotional responses (e.g., likability), trust-based judgments (e.g., credibility), and competence-based assessments (e.g., expertise), thereby offering a more comprehensive explanation of endorsement influence.

Finally, the study addresses a key limitation in prior literature that frequently treated social media platforms as interchangeable endorsement contexts. The findings demonstrate that millennials engage with platforms in distinct ways, seeking inspirational and lifestyle-oriented content on Instagram, spontaneous and authentic moments on Snapchat, and informational or expertise-driven cues on Twitter. These patterns underscore the importance of incorporating platform-specific user expectations and evaluative pathways into future theoretical development, particularly with respect to the balance between emotional and cognitive processing in digital persuasion.

### 6.2 Practical implications

From a managerial perspective, this study offers clear, evidence-based guidance for designing more effective sports celebrity endorsement strategies across social media platforms. A central practical contribution lies in demonstrating that endorsement success depends not only on selecting the “right” celebrity, but on aligning specific celebrity traits with the functional logic of each platform.

Likability emerged as a consistent driver of perceived product quality and purchasing intentions, particularly on visually oriented platforms. Brands targeting millennial consumers should therefore prioritize endorsers who convey warmth, relatability, and authenticity rather than relying solely on fame or performance credentials. Content strategies that showcase personal narratives, humor, and everyday moments are especially effective on Instagram and Snapchat, where emotional connection plays a dominant role. Interview insights confirm that behind-the-scenes and lifestyle-oriented posts increase trust and perceived realism, thereby strengthening endorsement impact.

Credibility represents another critical managerial consideration, particularly in environments where consumers actively assess authenticity. The findings indicate that frequent brand switching or inconsistent endorsements erode consumer trust, suggesting that long-term partnerships and visible product usage are more effective than short-term promotional campaigns. Snapchat's real-time and informal nature appears especially effective for signaling authenticity, making it a valuable channel for reinforcing credibility through unscripted or spontaneous content.

Expertise was shown to be particularly influential in shaping perceptions of product quality and purchase confidence, especially on Twitter. For technical, performance-related, or high-involvement products, brands should collaborate with athletes whose experience and knowledge are clearly relevant to the offering. The results suggest that detailed explanations, personal usage accounts, and informational content outperform image-based appeals in text-driven environments, reinforcing the need for message–platform congruence.

Physical attractiveness, while still relevant, was found to be insufficient as a standalone strategy. Its influence was strongest when combined with perceived authenticity, relevance, and demonstrated product fit, particularly in categories such as fitness, health, and wellness. This finding cautions managers against overreliance on aesthetic appeal and highlights the importance of holistic endorser–brand alignment.

Finally, a major practical contribution of this research is the demonstration that each platform requires a distinct endorsement strategy rather than content replication across channels. Instagram is best suited for emotionally engaging, visually rich storytelling; Twitter is optimal for expertise-driven, informational messaging; and Snapchat facilitates rapid trust-building through informal and authentic interactions. Marketers who adapt endorsement content, tone, and format to these platform-specific affordances are more likely to enhance perceived product quality and purchasing intentions among millennial consumers.

## 7 Limitations and future recommendations

This study has several limitations that should be considered when interpreting its findings. First, the sample size of 228 millennials may constrain generalizability. Future research should seek to include larger, more diverse samples. Second, reliance on online surveys and stratified sampling may have contributed to the overrepresentation of social media–active individuals with a strong interest in sports celebrities, potentially biasing results. Notably, 91.67% of respondents held a bachelor's degree, which may restrict the representativeness of findings across education levels. Self-reported data also involves risks of social desirability bias or misjudgment of personal behavior. Future studies could also incorporate behavioral tracking or observational tools to enhance accuracy.

Despite assessing multicollinearity (VIF < 5) to mitigate common method bias, the use of self-reported, cross-sectional surveys still raises concerns regarding potential common method variance, which should be considered when interpreting relationships among constructs. Additionally, the study context is limited geographically and culturally, as most participants are likely from similar backgrounds in Egypt, which may limit generalizability to other regions or cultures. The sample is relatively homogeneous with respect to age (23–41 years), education (mostly bachelor's degrees), and likely middle-income status, further constraining generalizability beyond comparable millennial populations.

Although Welch–Satterthwaite tests were employed as a complementary approach to PLS-MGA to account for unequal variances and sample sizes, formal measurement invariance testing (e.g., MICOM) was not undertaken. Therefore, cross-group comparisons should be interpreted with caution. Future research should prioritize conducting MICOM or other invariance assessments prior to MGA to support valid comparisons across platforms.

The study focuses exclusively on Instagram, Snapchat, and Twitter, which were historically prominent in sports endorsement research. However, other platforms such as TikTok and YouTube have become increasingly influential, and future research should consider cross-platform comparisons to capture a more comprehensive view of endorsement effectiveness in the current digital ecosystem.

The cross-sectional design limits the ability to draw causal inferences. Longitudinal or experimental designs are recommended to better assess changes over time. Future work should also explore different age cohorts (e.g., Gen Z, Gen X), compare platforms and industries, and further investigate how endorsement traits such as attractiveness or expertise are perceived across contexts. Such comparative approaches may strengthen the depth and applicability of endorsement research and support theory-building on cross-platform endorsement effectiveness.

## 8 Conclusion

This research clarifies how sports celebrities' personality traits—likability, expertise, credibility, and physical attractiveness—shape millennial consumers' reactions to product endorsements on Instagram, Snapchat, and Twitter. Using a mixed-methods approach, combining a survey of 228 active users with in-depth interviews of 12 participants, the study finds that each trait plays a distinct role: likability captures attention and fosters emotional engagement (ELM), expertise and credibility build trust and enhance perceived product quality (Source Credibility Theory; Cognitive Response Model), and physical attractiveness strengthens affective evaluation and endorsement memorability (ELM; Attitude-Toward-the-Ad Model).

The study underscores the importance of platform-specific strategies. Instagram seems particularly suited to promoting likability through images and stories, Twitter is more effective for demonstrating expertise and credibility, and Snapchat supports endorsements that feel spontaneous and authentic. The interviews reinforced these platform differences, showing that users tend to seek emotional connection on Instagram, reliable information on Twitter, and authenticity on Snapchat.

Perceived product quality emerged as a central mediating factor, with consumers forming evaluations based on how trustworthy, consistent, or relatable the celebrity appears. Qualitative insights revealed that these evaluations often occur quickly, relying on emotional shortcuts.

By combining quantitative and qualitative approaches, this study offers a more comprehensive understanding of how celebrity traits and platform dynamics relate to consumer behavior. The findings support and extend existing theories of celebrity endorsement, illustrating how Source Credibility Theory, ELM, Cognitive Response Model, and Attitude-Toward-the-Ad Model together help explain the mechanisms linking traits to perceived quality and purchase intentions. This dual approach adds theoretical depth and provides actionable guidance for marketers seeking to optimize engagement with millennials across diverse social media platforms.

## Data Availability

The original contributions presented in the study are included in the article/Supplementary Material, further inquiries can be directed to the corresponding author.

## Appendix A1 Constructs and adopted measurement scales

**Table A1:** Summary of constructs and adopted measurement scales.

Construct	Item	Source	Adaptation Notes
Personality Traits—Likability	I find the sports celebrity likable.	McCormick (63)	Wording modified to refer to social media posts on Instagram, Snapchat, or Twitter.
	The sports celebrity appears friendly and approachable on social media.		Adapted to fit the social media endorsement context.
	I enjoy the content shared by the sports celebrity on their social media accounts.		Wording aligned with Instagram, Snapchat, or Twitter content consumption.
Personality Traits—Expertise	The sports celebrity is knowledgeable about the endorsed product.		Adapted to emphasize online product knowledge.
	The sports celebrity provides useful information about the product in their social media posts.		Contextualized for social media endorsements.
	The sports celebrity seems competent in endorsing this type of product.		Slight rewording to reflect endorsement relevance.
Personality Traits—Credibility	The sports celebrity provides trustworthy information.		Context changed to Instagram, Snapchat, or Twitter.
	I can rely on the sports celebrity's social media recommendations.		Modified to reflect online consumer decision-making.
	The sports celebrity appears honest in their product endorsements online.		Wording adapted for digital endorsements.
Personality Traits—Physical Attractiveness	The sports celebrity is physically attractive.		No major changes.
	The sports celebrity's appearance captures my attention on social media.		Adapted to visual attention on Instagram, Snapchat, or Twitter feeds.
	I find the sports celebrity aesthetically appealing.		No major changes.
Perceived Product Quality	The product endorsed by the sports celebrity meets high-quality standards.	Akoglu & Özbek (64)	Adapted to reflect social media exposure.
	I believe the product performs as promised.		Minor wording adjustments.
	The product seems reliable based on the endorsement.		Aligned with online endorsement assessment.
Purchase Intention	I am likely to buy the product after seeing the sports celebrity endorse it on social media.	Priyankara et al. (65)	Contextualized for social media endorsements.
	I would consider purchasing this product based on the sports celebrity's online endorsement.		Adapted to match online decision-making.
	I would recommend this product to others based on the social media endorsement.		Added “based on social media endorsement.”

1. All items were adapted to the social media context (Instagram, Snapchat, Twitter) to accurately capture respondents' perceptions of sports celebrity traits and endorsements across platforms.

2. Purchase intention items reflect both likelihood of purchase and willingness to recommend, consistent with the behavioral patterns identified in the mixed-methods findings.

3. All items were measured on a 5-point Likert scale (1 = Strongly Disagree to 5 = Strongly Agree).

## Appendix A2 Semi-structured interview guide

**Table A2:** Interview guide with main questions and probes.

Section	Main Questions	Probes
1. General Engagement and Perception	How often do you follow sports celebrities on your primary platform? What types of posts capture your attention most (e.g., videos, images, stories, written posts)?	Influence of behind-the-scenes content on perceptions of the celebrity.
2. Likability	What makes a sports celebrity likable to you on your primary platform? Can you describe posts or behaviors that strengthened your emotional connection?	Personal stories, casual moments, interactions with fans.
3. Expertise	How do you recognize a sports celebrity as knowledgeable or expert about products on your primary platform? Can you recall a situation where their expertise affected your perception?	Detailed explanations, performance insights, product reviews.
4. Credibility/Trust	What factors make you trust or doubt a sports celebrity's endorsement? Can you give examples of when the celebrity felt authentic versus insincere?	Consistency, long-term partnerships, transparency, platform-specific content.
5. Physical Attractiveness	To what extent does a celebrity's physical appearance influence your perception of endorsed products? Can you share an example where attractiveness combined with performance influenced your purchase intention?	Discipline, fitness, lifestyle relevance, platform context.
6. Platform-Specific Experience	How does your primary platform shape your perception of celebrity traits? Are there differences in how you respond to the same celebrity across Instagram, Twitter, or Snapchat?	Visual features, storytelling style, engagement mechanisms (comments, polls, stories).
7. Purchase Intention	Have you ever purchased a product because of a celebrity endorsement? If yes, describe the experience. How do likability, expertise, credibility, and physical attractiveness influence your purchase decisions?	—
8. Closing	Is there anything else about following sports celebrities that influences your opinions or purchase decisions? Any final comments on how platform or content type affects your perceptions?	—

## Appendix A3 Examples of coding, categories, and themes from interviews

**Table A3:** Examples of how raw participant statements were coded, grouped into categories, and synthesized into overarching themes.

Raw Statement	Initial Code	Category/Axial Code	Theme
“I follow a few athletes who post behind-the-scenes stuff—family dinners, workouts, even silly moments. That's when I start to feel like I know them. It's not just a brand talking—it's them.”	Behind-the-scenes content	Emotional connection	Authentic Engagement Enhances Likability
“If I like the person, I'm more open to what they're promoting—even if I hadn't considered that product before.”	Liking influences openness		
“Even short stories or candid clips make me feel like I understand their personality. That makes me trust their recommendations more.”	Casual, relatable content		
“When a footballer breaks down how their boots help them pivot better or gives technical feedback on a supplement—it sticks. That's what I want to hear.”	Product knowledge demonstration	Expertise display	Expertise Must Be Contextual and Demonstrated
“Twitter is where I go for insights. I don't care if they look good—I care if they know what they're talking about.”	Platform-specific expertise		
“I trust celebrities who show me exactly how they use a product. Their knowledge matters more than appearance.”	Demonstrated usage		
“If I see someone pushing different brands every week, I stop trusting them. But when it's someone who's been with the same brand for a while—and shows how they use it—I take it seriously.”	Consistency in endorsement	Trust & credibility	Trust and Credibility Are Built Through Consistency
“Snapchat feels more real. You catch them off guard sometimes. That's where I judge if they actually believe in the product.”	Candid content fosters trust		
“I look at how consistent their endorsements are. If it matches their values and performance, I trust them more.”	Value alignment		
“Yeah, looks catch attention—but if that person clearly trains hard and maintains discipline, I respect that. It tells me their product might reflect that same quality.”	Attractiveness + performance	Contextual relevance	Physical Attractiveness Is Context-Dependent and Aspirational
“Attractiveness matters, but only when it connects with their performance. I'd buy protein powder from someone who looks like they use it—not just someone with a filter.”	Visual appeal + relevance		
“On Instagram, attractiveness draws me in, but performance and authenticity keep me engaged.”	Looks + authenticity		
